# Rationally Tailoring Superstructured Hexahedron Composed of Defective Graphitic Nanosheets and Macropores: Realizing Durable and Fast Potassium Storage

**DOI:** 10.1002/advs.202205234

**Published:** 2022-11-24

**Authors:** Fei Yuan, Conghao Shi, Yanan Li, Jian Wang, Di Zhang, Wei Wang, Qiujun Wang, Huan Wang, Zhaojin Li, Bo Wang

**Affiliations:** ^1^ Hebei Key Laboratory of Flexible Functional Materials School of Materials Science and Engineering Hebei University of Science and Technology Shijiazhuang 050000 China; ^2^ School of Metallurgical and Ecological Engineering University of Science and Technology Beijing Beijing 100083 China

**Keywords:** anode, defect‐rich, graphitic structure, macropores, potassium‐ion battery

## Abstract

Multipores engineering composed of micro/mesopores is an effective strategy to improve potassium storage performance via providing enormous adsorption sites and shortened ions diffusion distance. However, a detailed exploration of the role played by macropores in potassium storage is still lacking and has been barely reported until now. Herein, a superstructure carbon hexahedron (DGN‐900) is synthesized using poly tannic acid (PTA) as precursor. Due to the spatially confined two‐step local contraction of PTA along different directions and dimensions during pyrolysis, defective nanosheets with macropores are formed, while realizing a balance between defects content and graphitization degree by regulating temperature. The presence of macropores is conducive to accelerating electrolyte ions rapid infiltration within electrode, and its pore volume can accommodate electrode structure fluctuation upon cycling, while the most suitable ratio of defects to graphitic provides rich ions adsorption sites and sufficient electrons transfer channels, simultaneously. These advantages enable a prominent electrochemical performance in DGN‐900 electrode, including high rate (202.9 mAh g^−1^ at 2 A g^−1^) and long cycling stability over 2000 cycles. This unique fabrication strategy, that is, defects engineering coupled with macropores structure, makes fast and durable potassium storage possible.

## Graphical abstract

Due to the spatially confined two‐step local contraction of poly tannic acid (PTA) along different directions and dimensions during carbonization, defective nanosheets with macropores are formed, while realizing a balance between defects content and graphitization degree by regulating temperature. All of which endow the fast transfer ability of both ions/electrons, and alleviate volume fluctuation, leading to improved capacity and cyclability.

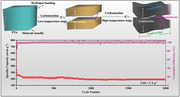

## 1 Introduction

Commercialized lithium‐ion batteries (LIBs) are the most commonly used energy storage devices in the past two decades and are currently applied in electric vehicles,^[^ ^1^, ^2^ ^]^ due to their high energy density and long cycle life, etc. However, based on the long‐term sustainable perspective, limited lithium resources (0.0017 wt% in the earth crust) and ever‐increasing cost will inevitably hamper its further development,^[^ ^3^, ^4^ ^]^ which prompts researchers to explore alternatives to LIBs urgently. Particularly, considering resource abundance (1.5 wt%), cost‐effectiveness (e.g., K_2_CO_3_), and similar working mechanism to LIBs,^[^ ^3^, ^5^, ^6^ ^]^ potassium‐ions batteries (PIBs) have attracted enormous attention in recent years, and are theoretically demonstrated to be able to provide high voltage platform and high energy density, resulting from a low redox potential of K/K^+^ versus standard hydrogen electrode (−2.93 V, that is closed to −3.04 V of Li/Li^+^).^[^ ^1^, ^7^, ^8^ ^]^ Accordingly, a large number of related studies have been reported, especially for anode materials, which significantly promote the development of PIBs.

Among all the anodes, carbonaceous materials are highly preferable and have been extensively investigated because of their excellent electrical conductivity and chemical stability.^[^ ^9^, ^10^, ^11^ ^]^ Nevertheless, K‐ions with a larger size (1.38 Å for K^+^ versus 0.76 Å for Li^+^) can induce more distinct volume variation of the host electrode and sluggish kinetics for solid‐state diffusion during intercalation/de‐intercalation process,^[^ ^12^, ^13^, ^14^ ^]^ eventually leading to serious capacity decay and inferior rate. Defect engineering in carbon matrix has been proven to be an effective strategy to resolve these issues, and simultaneously contributes to breaking through theoretical capacity to a large extent.^[^ ^9^, ^15^ ^]^ This is mainly due to the formed C−C sp3 defects being able to provide efficient K^+^ diffusion pathways,^[^ ^16^, ^17^ ^]^ while active surfaces or voids are conducive to adsorbing numerous K‐ions,^[^ ^18^, ^19^ ^]^ leading to a high capacitive contribution. Besides that, previous reports have demonstrated that micropores (<2 nm) can function as K‐ions storage sites and the suitable pore volume is an ideal buffer space for electrode structure change, while mesopores (20–50 nm) are favorable for reducing ions diffusion distance, resulting in enhanced reaction kinetics.^[^ ^8^, ^20^ ^]^ However, a comprehensive exploration of macropores (>50 nm) for potassium storage is still lacking up to now. As we all know, macropores can act as electrolyte reservoir to accelerate electrolyte ions infiltration,^[^ ^21^, ^22^ ^]^ which not only in return moderates the concentration polarization, but boosts rate performance. Meanwhile, benefiting from the carbon nano‐wall between two adjacent macropores, the evenly distributed macropores configurations can well decrease solid‐state diffusion length of K‐ions to nanometer scale,^[^ ^22^ ^]^ endowing improved ions transfer ability, especially at a high current density. On account of this, coupling the structural advantages of defect engineering with the function of macropores into one material is extremely attractive for tailoring carbon anode with high capacity, rate, and cyclability.

Herein, we elaborately develop a superstructured hexahedron composed of defective graphitic nanosheet and macropores via a hydrothermal reaction and subsequent carbonization process (denoted as DGN‐900). A series of structural characterizations indicate that the presence of rich defects provides sufficient adsorption sites for K‐ions storage, while plenty of continuous graphite domains contribute to fast electrons transfer, thus endowing a high conductivity. Not only that, the macropores formed in wrinkled graphitic carbon nanosheets facilitate the fast permeation of electrolyte, and these macropores can act as buffer space to accommodate volume change during insertion/extraction of K‐ions, ensuring fast reaction kinetics and durable structure stability. All these together make DGN‐900 a promising anode for PIBs, including high reversible capacity of 259 mAh g^−1^ at 0.5 A g^−1^ and impressive rate capability at 2 A g^−1^ (202.9 mAh g^−1^), accompanied by an excellent lifespan over 2000 cycles with a Coulombic efficiency of nearly 100%.

## 2 Results and Discussion

### 2.1 Synthesis and Structural Analysis of Materials

**Figure 1:**
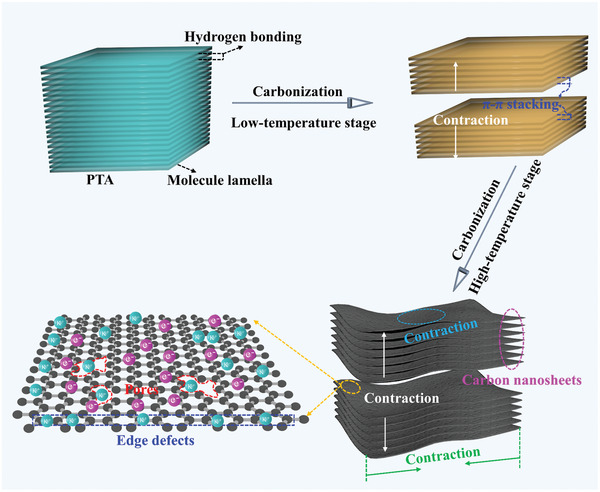
Schematic illustration of layered subunit. The number of lamellas in one nanosheet is for illustration only.

Figure S1 (Supporting Information) displays the synthetic process of target products, in which tannic acid (TA) and F127 were used as raw materials. Briefly, poly tannic acid precursors (PTA) were firstly synthesized via hydrothermal reaction under an alkaline condition, and then this PTA was annealed at a high temperature. According to previous report,^[^ ^23^ ^]^ PTA with a lamellar structure would trigger a spatially confined two‐step local contraction during pyrolysis process. The former happened at a low temperature along the longitudinal direction of PTA (**Figure** **1**), which was primarily induced by the formed π–π stacking between adjacent molecular layers and the dehydration between adjacent lamellas,^[^ ^23^ ^]^ thus enabling the formation of nanosheets; while the latter occurred at a higher temperature and was deeply determined by carbonization process in three dimensions,^[^ ^23^ ^]^ and through in‐plane confined contraction, the superstructure carbons comprise defective nanosheets with macropores were ultimately established. Because of these structure features, the optimized sample is believed to deliver a superior electrochemical performance as an anode for PIBs.

**Figure 2:**
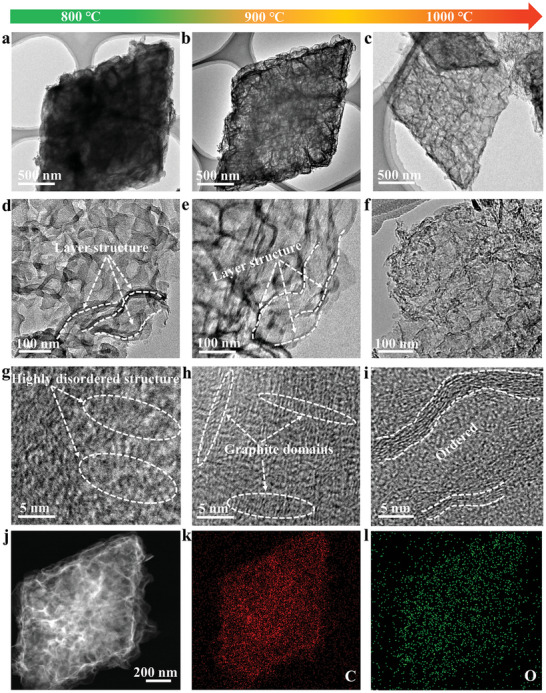
The morphology transformation with the increase of pyrolysis temperature displayed by transmission electron microscopy (TEM) images. a,d) TEM and g) high‐resolution TEM (HRTEM) images for DGN‐800. b,e) TEM and h) HRTEM images for DGN‐900. c,f) TEM and i) HRTEM images for DGN‐1000. j) Annular dark‐field STEM image and k,l) corresponding elemental mapping of DGN‐900.

As shown in Figure S2 (Supporting Information), transmission electron microscopy (TEM) image indicates that PTA has a visible solid‐rod structure with rhombus‐like across‐section morphology. After pyrolysis at different temperatures (denoted as DGN‐800/900/1000), rhombus‐shaped profile structures are well retained while the solid structure configurations are continuously discomposed, accompanied by the formation of pore structures, as evidenced by TEM images in **Figure** **2**a–c. Through field‐emission scanning electron microscopy (SEM) images of DGN‐900 in Figure S3 (Supporting Information), one can see that these rhombus‐like across‐section morphologies are actually hexahedron comprised of multilayer wrinkled nanosheets, which is further confirmed by magnified TEM images in Figure 2d–f. Notably, with the increase of temperature, the layer structure gradually becomes inconspicuous until it disappears at 1000 °C, meaning the reduction of distance between nanosheet layers. This is further supported by the study of atomic force microscopy (AFM) in Figure S4 (Supporting Information), where the average thickness of these three samples is found to decrease in the order of DGN‐800 (15.3 nm) > DGN‐900 (8.6 nm) > DGN‐1000 (4.2 nm). In the high‐resolution TEM (HRTEM) image (Figure 2g–i), we can clearly see that the microstructure unit of these three samples changes from highly disordered structure in DGN‐800 to graphite‐domains in DGN‐900 and then to ordered structure in DGN‐1000, suggesting a distinct improved the degree of graphitization as the temperature increases. Meanwhile, this result is also highly responsible for a good conductivity, as confirmed by the result of electronic conductivity measurements (Figure S5, Supporting Information). Energy‐dispersive X‐ray spectroscopy (EDS) mapping (Figure 2j–l) illustrates that DGN‐900 is mainly composed of C and O elements. As a comparison, TEM image of TA derived carbon at 900 °C (named as TA‐C) was also collected in Figure S6 (Supporting Information), one can see that TA‐C has a sheet‐like morphology with dense structure, which proves that the PTA precursor has a decisive effect on the formation of wrinkled nanosheets with pores.

**Figure 3:**
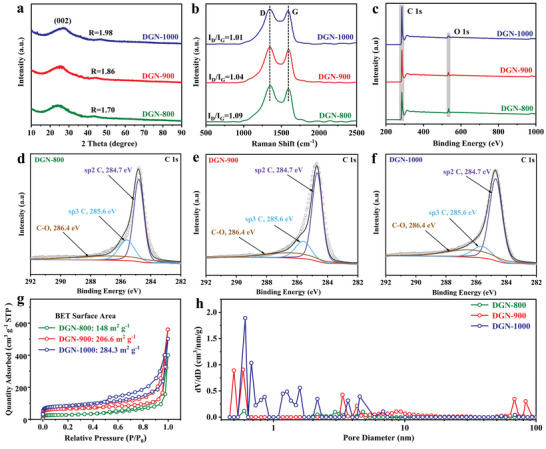
a) X‐ray diffraction (XRD) patterns, b) Raman spectra, and c) X‐ray photoelectron spectroscopy (XPS) survey of DGN‐800, DGN‐900, and DGN‐1000. d–f) High‐resolution C 1s XPS spectra of DGN‐800, DGN‐900, and DGN‐1000, respectively. g) Nitrogen adsorption–desorption isotherms and h) pore size distribution of DGN‐800, DGN‐900, and DGN‐1000 electrodes based on the density functional theory approach.

The crystal structures of DGN‐800/900/1000 and TA‐C were investigated by X‐ray diffraction (XRD) patterns. As observed in Figure S7 (Supporting Information), TA‐C exhibits a broad diffraction peak at around 23.9°, demonstrating a typical disordered structure. The *R*‐value, introduced by Dahn and co‐workers,^[^ ^24^ ^]^ is usually employed as an indicator to reflect the order or graphitization degree of carbon materials. Through the calculation process in Figure S8 (Supporting Information), it is found that *R*‐values increase from 1.70 to 1.98 with increasing carbonization temperature (**Figure** **3**a), which means an enhanced graphitic structure, agreeing well with the observation of HRTEM images. Through Raman spectra in Figure 3b, two obvious peaks at 1353 and 1596 cm^−1^ are observed, indexing to D‐band and G‐band of carbon matrix,^[^ ^25^, ^26^ ^]^ respectively. The integrated peak intensity (*I*_D_/*I*_G_) ratio can intuitively unveil the degree of structure defects, and as a result DGN‐900 has a medium *I*_D_/*I*_G_ value (1.04) in comparison with DGN‐800 (1.09) and DGN‐1000 (1.01), demonstrating its suitable defects and graphitic domain units. Besides, the elemental composition and chemical state of these three samples were explored by X‐ray photoelectron spectroscopy (XPS). It can be known that DGN‐800/900/1000 mainly consists C and O elements (Figure 3c), and the corresponding atomic contents are summarized in Figure S9a (Supporting Information). Considering XPS is a surface technique with a penetration depth of several nanometers, so the bulk elemental composition was measured by combustion elemental analysis. Evidently, the C‐content depicted in Figure S9b (Supporting Information) is lower compared to XPS result for these three samples, demonstrating that the distribution of O is more concentrated in the inner of carbon matrix. This result reveals that the obtained C content by XPS is relatively higher than that in the whole carbon matrix, in accordance with the previously reported works.^[^ ^4^, ^5^ ^]^ Moreover, the detected O is supposed to improve the wettability of electrode materials and increase the utilization ratio of active surface,^[^ ^10^, ^13^ ^]^ thus favoring a good rate and capacity. As displayed in Figure 3d–f, the high resolution XPS spectrum of C 1s can be deconvoluted into three peaks at 284.7, 285.6, and 286.4 eV,^[^ ^27^, ^28^ ^]^ corresponding to sp2 C, sp3 C, and C−O, respectively. The detected sp3 C represents the intrinsic defects of carbon matrix,^[^ ^28^ ^]^ and it is easy to observe that the integral area ratio of sp3 C decreases in the order of DGN‐800 > DGN‐900 > DGN‐1000, indicating a decreased defects proportion. This result is in good agreement with the analysis of HRTEM, XRD, and Raman, and at the same time confirms the fact that DGN‐900 has the optimal defects‐to‐graphitic ratio. Figure 3g presents N_2_ adsorption–desorption measurement results of these three samples, in which Brunauer–Emmett–Teller (BET) specific surface area (SSA) are 148, 206.6, and 284.3 m^2^ g^−1^, respectively. As depicted in Figure 3h, all samples possess micro/mesopores features, and pore volumes increase significantly with increasing pyrolysis temperature (Table S1, Supporting Information), which well explains what causes the difference in SSA. More importantly, it should be pointed out that there is a certain amount of macropores in DGN‐900, and average pore diameter is about 83 nm. Compared to DGN‐800 without macropores and DNG‐1000 with slightly macropores, the macropores volume of DGN‐900 is as high as 25.2 cm^3^ g^−1^ (Table S1, Supporting Information), signifying that carbonization temperature plays a key role in determining the formation of macropores. Concretely, some large radicals cannot be completely decomposed at a relatively low temperature,^[^ ^29^ ^]^ so DGN‐800 has no macropores structure. With increasing temperature to 1000 °C, macropores structure will mostly collapse due to the shrinkage of carbon nanosheets, resulting in the reduction of their number and volume. Therefore, the macropores observed in DGN‐900 are believed to be conducive to avoiding sluggish kinetics and structure instability during charge storage process, endowing enhanced rate and cycle life. As for TA‐C (Figure S10, Supporting Information), its SSA (only 20.2 m^2^ g^−1^) is much lower than that of DGN‐900, indicating that the PTA rather than TA is important for exposing the pore structures. Subsequently, thermogravimetric analysis (TGA) was used to investigate the pyrolysis behaviors of PTA. As shown in Figure S11 (Supporting Information), an obvious mass loss of 11.1% before 230 °C can be ascribed to the thermal condensation of interplane OH groups, and then there is almost no weight loss between 230 °C and 400 °C due to the generation of π–π stacking interaction.^[^ ^23^ ^]^ The further thermal decomposition of PTA with mass loss of 32.6% is observed at 400–500 °C, which is likely ascribed to the disappearance of π–π stacking interaction in this temperature range.^[^ ^23^ ^]^ Massive weight loss after 500 °C may be the result of the decomposition of some organic molecules at a higher temperature, which further well supports the macropores evolution mechanism.

### 2.2 Electrochemical Performance of Potassium‐Ion Half Cell

**Figure 4:**
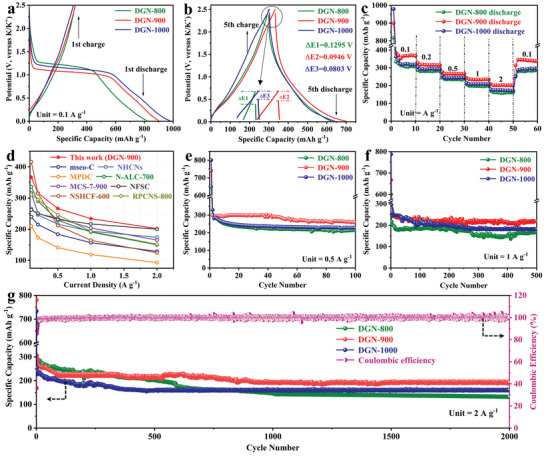
a) The first and b) fifth charging–discharging curves of DGN‐800, DGN‐900, and DGN‐1000 at 0.1 A g^−1^. c) Rate capabilities of DGN‐800, DGN‐900, and DGN‐1000 at different current densities. d) Comparison of the rate performance of the DGN‐900 with other anodes. e,f) Cycling performance and g) long cycling stability of DGN‐800, DGN‐900, and DGN‐1000, respectively.

As aforementioned, DGN‐900 features rich macropores and good defects‐to‐graphitic ratio, all of which are believed to account for excellent potassium storage performance. So, a K‐half cell was assembled using DGN‐900 as anode, and its electrochemical performance was first evaluated by galvanostatic charge–discharge measurements. As shown in **Figure** **4**a, the initial Coulombic efficiency (ICE) of DGN‐900 is 35.6%, which is better than DGN‐1000 (31.3%) but lower than DGN‐800 (38.8%). Such a large initial active potassium loss in DGN‐900 may be the result of the decomposition of electrolyte and formation of solid electrolyte interphase (SEI) film,^[^ ^30^, ^31^ ^]^ in line with the observation of cyclic voltammetry (CV) (Figure S12, Supporting Information). Furthermore, the ICE is found to be tightly related to SSA value as indicated in Figure S13a (Supporting Information), and decreases linearly with the increase of SSA. In Figure S13b (Supporting Information), it can be learned that there is no straightforward correlation between ICE and *I*_D_/*I*_G_. These results reveal that it is SSA rather than defects content that influences ICE, which is explained by the fact that a lower SSA can reduce the excessive depletion of electrolyte caused by undesirable side reactions. However, according to previous report,^[^ ^32^ ^]^ a low ICE can be well improved by employing 1,2‐dimethoxyethane (DME) based electrolyte with a higher concentration or 1,2‐diethoxyethane (DEE) based electrolyte with decreased desolvation energy. As displayed in Figure 4b, voltage drop decreases from 0.1295 V in DGN‐800 to 0.0946 V in DGN‐900 and then to 0.0803 V in DGN‐1000, further illustrating an enhanced conductivity.^[^ ^33^ ^]^ Figure 4c shows rate performances of these three samples, in which the average specific capacities of DGN‐900 are 370.8, 316.3, 266.3, 233.8, and 202.9 mAh g^−1^ at 0.1, 0.2, 0.5, 1, and 2 A g^−1^, respectively, markedly outperforming its counterparts DGN‐800 and DGN‐1000. The discharge–charge curves in Figure S14 (Supporting Information) once again highlight the excellent rate capability of DGN‐900 by keeping a constant curve shape at different current densities. Notably, the rate performance of electrode materials is usually codetermined by its ions transfer and electronic conductivity, so here exceptional rate capability observed in DGN‐900 is ascribed to its well‐connected macropores and rich defects that greatly accelerate ions transfer, and good conductivity enabled by plenty of continuous graphitic domains. Based on this, the rate capacities of DGN‐900 electrode are superior to some previously reported carbon anodes as well,^[^ ^5^, ^14^, ^15^, ^34^, ^35^, ^36^, ^37^, ^38^ ^]^ as shown in Figure 4d. Cycling performance of DGN‐900 was also tested at 0.5 and 1 A g^−1^, respectively. The discharge capacity of DGN‐900 is 259 mAh g^−1^ after 100 cycles at 0.5 A g^−1^ (Figure 4e), which is higher than those of DGN‐800 (214.6 mAh g^−1^) and DGN‐1000 (228 mAh g^−1^). Even cycled at 1 A g^−1^ over 500 cycles (Figure 4f), DGN‐900 still delivers a high capacity of 218.9 mAh g^−1^, while DGN‐800 and DGN‐1000 only maintain 169.7 and 182.1 mAh g^−1^, respectively. As for long‐term cycling stability, DGN‐900 realizes a superior reversible capacity of 193.5 mAh g^−1^ with close to 100% Coulombic efficiency even after 2000 cycles (Figure 4g), which is far above most materials reported in the previous literature (Table S2, Supporting Information); however, both DGN‐800 and DGN‐1000 electrodes display an obvious capacity decay and ultimately deliver 131.5 and 160.4 mAh g^−1^, respectively. The reason for fast capacity fading of DGN‐1000 electrode before 160 cycles at 2 A g^−1^ is likely due to its increased graphitization degree and decreased defect sites, and the stabilized capacity during subsequent cycling is ascribed to its obvious macropores volume, which affords buffer space to accommodate electrode structure fluctuation. For DGN‐800, although it has the highest structure defects, its low SSA and poor pore volume tend to cause less exposed reaction sites, especially the lack of macropores that effectively offers buffer space, resulting in the lowest capacity and fastest decay. Undoubtedly, due to the lack of sufficient pore configurations caused by a dense structure, TA‐C owns inferior rate (Figure S15, Supporting Information) and poor cyclability (Figure S16, Supporting Information). The results above reasonably demonstrate that DGN‐900 with comprehensive merits, such as the most suitable ratio of defects‐to‐graphitic and well‐developed macropores in nanosheets, can provide fast ions/electrons transport capability, rich K‐ions storage sites, and adequate buffer space for volume change, all these together endow higher capacity, superior rate capability, and advanced durability.

### 2.3 Kinetics and K‐Storage Mechanism

**Figure 5:**
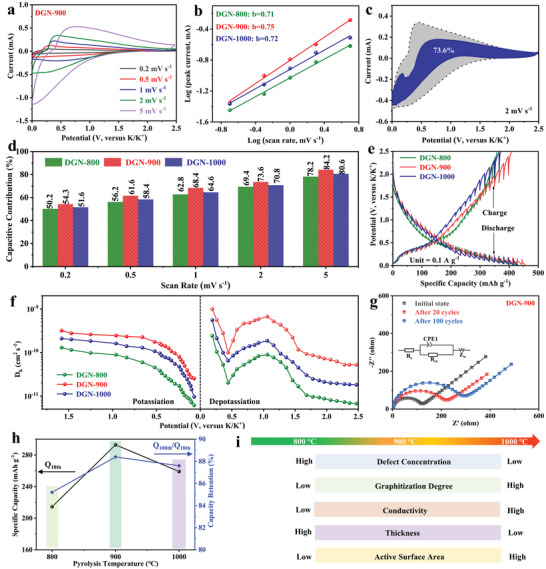
a) Cyclic voltammetry (CV) curves at different scan rate. b) *b*‐values. c) Surface capacitive contribution of the DGN‐900 at the scan rate of 2 mV s^−1^. d) Capacitive contribution of DGN‐800, DGN‐900, and DGN‐1000 at various scan rates. e) Galvanostatic intermittent titration technique (GITT) profiles. f) K‐ion diffusion coefficient. g) Nyquist plots of DGN‐900 before and after cycling. h) The variation of reversible capacity and capacity retention with pyrolysis temperature. i) Schematic illustrations of the physical parameters with pyrolysis temperature.

The K‐ions storage mechanism was further studied via CV curves tests of DGN‐800/900/1000 at different scan rates, as indicated in **Figure** **5**a and Figure S17 (Supporting Information). Generally, the total stored charge of the CV curves is mainly composed of two parts, that is, surface capacitive behavior and diffusion process, which can be quantitatively investigated by a power‐law formula,^[^ ^39^, ^40^ ^]^ that is, *i* = a*v* ^b^, in which *i* is the peak current and *v* is the scan rate. The *b* value usually is employed as an indicator to reflect capacitive behavior (*b* = 1) or diffusion process (*b* = 0.5), and it can be extracted by the slope of the log(*i*) versus log(*v*) plot.^[^ ^41^, ^42^ ^]^ As shown in Figure 5b, the calculated *b* values are 0.71, 0.75, and 0.72, respectively, suggesting a more dominant capacitive process in DGN‐900. Moreover, capacitive contribution to total capacity can be further quantified based on the formula of *i* = *k*_1_ *v* + *k*_2_ *v* ^1/2^,^[^ ^43^, ^44^ ^]^ where *k*_1_ *v* stands for the capacitive reaction. As depicted in Figure 5c, the capacitive contribution is calculated as 73.6% at fixed scan rate of 2 mV s^−1^, and this value is expanded to 84.2% at 5 mV s^−1^ (Figure 5d). Note that DGN‐900 always exhibits a higher capacitive contribution ratio at each scan rate compared to DGN‐800 and DGN‐1000. To explore reaction kinetics in‐detail, galvanostatic intermittent titration technique (GITT) was used to investigate K‐ions diffusion coefficient (*D*_K_). By applying a series of pulse current at 0.1 A g^−1^ for 10 min and relaxation process for 1 h, the obtained GITT curves are presented in Figure 5e. On the basis of calculation process displayed in Figure S18 (Supporting Information), we can obviously see that *D*_k_ values in DGN‐900 are higher than those of DGN‐800 and DGN‐1000 electrodes at all the potential (Figure 5f), revealing the faster K‐ions diffusion kinetics. Electrochemical impedance spectroscopy (EIS) results further emphasize the superior dynamic behavior in DGN‐900 (Figure 5g and Figure S19, Supporting Information), where it exhibits a smaller charge transfer impedance at each stage in contrast to DGN‐800 and DGN‐1000 electrodes. Based on the systematical analysis above, we can conclude that the presence of unique macropores and suitable defects in DGN‐900 accelerate the infiltration of the electrolyte ions along with provide sufficient K‐ions adsorption sites, and a large number of continuous graphite domains function as fast electrons transfer channels, consequently leading to excellent rate capacities. Combining with Figure 5h,i, evidently, regulating carbonization temperature is very important to achieve a balance between defects and conductivity, because only a suitable defects‐to‐graphitic ratio can make full use of the synergistic effect of fast ions diffusion/storage and electrons transfer, thus enabling the highest capacity and capacity retention, as displayed in DGN‐900.

**Figure 6:**
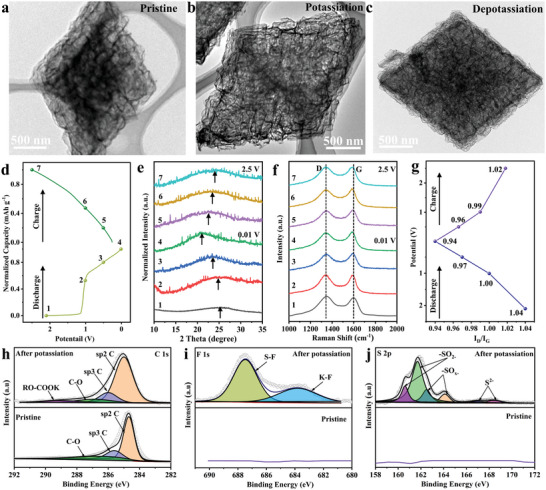
Transmission electron microscopy (TEM) images of DGN‐900 at the original state (a), after full potassiation (b), and after full depotassiation (c). d) Test potentials for ex situ characterizations. e) Ex situ X‐ray diffraction (XRD) of DGN‐900 at different stages. f) Ex situ Raman spectra and g) corresponding *I*_D_/*I*_G_ values. High‐resolution X‐ray photoelectron spectroscopy (XPS) spectra of h) C 1s, i) F 1s, and j) S 2p characteristic peaks for DGN‐900 before and after cycling (the cells were cycled at 0.1 A g^−1^).

The structural and composition evolution of DGN‐900 upon potassiation/depotassiation was systematically studied by ex situ TEM, ex situ Raman, ex situ XRD, etc. As observed in **Figure** **6**a–c, the rhombus‐like morphologies have hardly changed throughout the discharge–charge process, even after 2000 cycles, this structure is still well maintained apart from a slight pulverization (Figures S20 and S21, Supporting Information), intuitively highlighting a cycling durability. In Figure 6d,e, the (002) diffraction peak hardly changes at voltage above 1.0 V, while obviously shifts to a lower angel at voltage below 0.5 V, once again confirming a mixed potassium storage mechanism of DNG‐900, namely capacitive adsorption at high potential region and intercalation process at low potential region. At the end of charge, the position of (002) peak can nearly recover to its pristine level, indicating a high reversibility of DGN‐900 electrode. Another interesting phenomenon is found in ex situ Raman patterns (Figure 6f,g), in which *I*_D_/*I*_G_ value decreases from 1.04 to 0.94 during the whole discharge process, which can be explained by the adsorption of K‐ions at defect sites and vacancies.^[^ ^45^, ^46^ ^]^ After fully charged to 2.5 V, the *I*_D_/*I*_G_ value (1.02) that is very close to the initial state further confirms a prominent reversibility, in accordance with the analysis of ex situ XRD. After potassiation, the whole C1s peak slightly shifts to higher binding energy, and an indistinct new peak (RO−COOK) appears at about 289.9 eV (Figure 6h), which is due to the formation of C−K species and slight decomposition of carbonate solvents,^[^ ^37^ ^]^ respectively. The clearly observed K−F and S−F species (Figure 6i) as well as sulfide‐based compounds (Figure 6j) signify the decomposition of potassium bis(fluoroslufonyl) imide (KFSI),^[^ ^47^ ^]^ thereby ensuring the formed SEI layer is mainly composed of inorganic rather than organic species, accounting for a good interfacial stability. Besides, thanks to residual K‐ions in carbon matrix and SEI film,^[^ ^37^, ^48^, ^49^ ^]^ K 2p peak still exists after full depotassiation (Figure S22, Supporting Information), this is also the reason for the loss of initial active K‐ions.

### 2.4 Electrochemical Performance of Potassium‐Ion Full Cell

**Figure 7:**
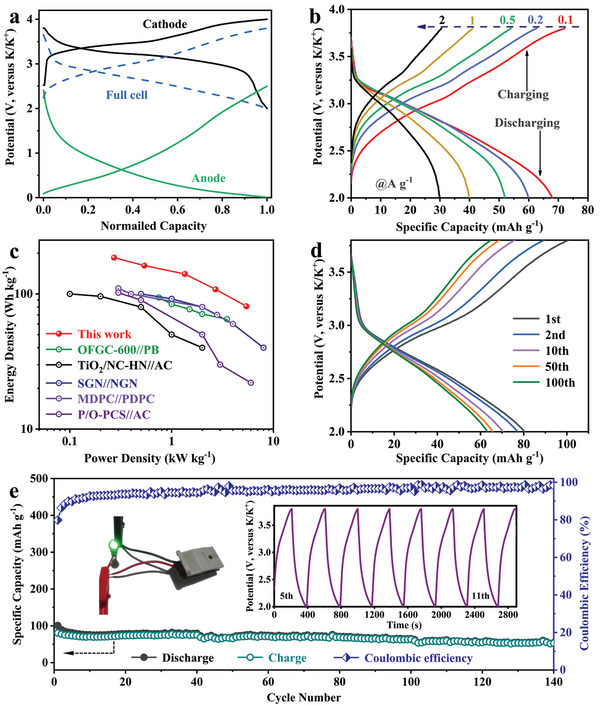
a) Normalized charge–discharge profiles of half/full cells. b) Charge–discharge profiles of DGN‐900//potassium Prussian blue (KPB) full cell at different current densities from 0.1 to 2 A g^−1^. c) Ragone plots of DGN‐900//KPB full cell compared with the reported representative potassium‐ions batteries (PIBs) and potassium‐ion hybrid capacitors. d) Charge–discharge profiles and e) cycling performance of DGN‐900//KPB full cell at 0.1 A g^−1^. Inset: the photograph of light‐emitting diode (LED) bulbs powered by our DGN‐900//KPB device and charge–discharge curves.

To evaluate the practical application of DGN‐900, a K‐full cell was successfully assembled using prepotassiated DGN‐900 and potassium Prussian blue (KPB) as anode and cathode, respectively. As shown in Figure S23 (Supporting Information), the obtained KPB owns a nanosized morphology, and its cycling performance and charge–discharge curves are further presented in Figure S24 (Supporting Information). Based on the normalized charge–discharge curves of the full and half cells observed in **Figure** **7**a, the matched DGN‐900//KPB full cell can be operated normally in the voltage range of 2–3.8 V. The charge–discharge curves at different current densities are shown in Figure 7b, and the calculated energy density is as high as 185.6 Wh Kg^−1^ at 0.1 A g^−1^ (all calculated according to the weight of anode). In addition, compared to previously reported PIBs and potassium‐ion hybrid capacitor,^[^ ^37^, ^47^, ^50^, ^51^, ^52^ ^]^ this DGN‐900//KPB full cell still has superior energy density at different power densities as indicated in Ragone plots in Figure 7c. As the cycling proceeds, the charge–discharge curves shape is greatly maintained (Figure 7d), demonstrating a good cyclability. It is evident that the Coulombic efficiency can rapidly increase to above 90% after five cycles, and reaches nearly 98% over 140 cycles (Figure 7e), indicating the stable working of this full cell. Moreover, the fully charged DGN‐900//KPB can successfully power a light‐emitting diode (LED), emphasizing its feasibility in the application of portable electronic devices.

## 3 Conclusions

In summary, we elaborately develop a superstructured carbon hexahedron using PTA as precursors. During the pyrolysis process, the PTA with a lamellar structure can induce a spatially confined two‐step local contraction, eventually resulting in the formation of defective graphitic nanosheets and macropores. The well‐interconnected macropores can facilitate the permeation of electrolyte ions and function as volume buffer space, which endows fast ions transfer capability and improved structural integrity. Besides, through the modulation of carbonization temperature, the balance between defects content and the degree of graphitization is realized, which not only favors the fast diffusion of K‐ions enabled by abundant active sites, but also contributes to a good electronic conductivity with the help of the plenty of continuous graphite domains. Accordingly, the optimized DGN‐900 delivers a high capacity, superior rate, and prolonged cycle lifespan by retaining 193.5 mAh g^−1^ at 2 A g^−1^ over 2000 cycles. This work comprehensively demonstrates the synergistic effect of macropores and the most suitable ratio of defects to graphitic on potassium storage performance, and may provide a reference for the preparation of other advanced carbon anodes.

## 4 Experimental Section

### Synthesis of Defective Graphitic Nanosheets (DGN‐T, *T* = 800 °C, 900 °C, and 1000 °C)

In a typical synthesis process, 0.2 g of F127 was dissolved in 46 mL of water and 8 mL of ethanol at room temperature (25 °C). Then, 0.1 mL of ammonium hydroxide was added in the above solution. After stirring for 1 h, 0.2 g of TA was added in the system and the mixture was stirred for another 24 h. The obtained mixture was transferred to an autoclave and hydrothermally treated for 24 h at 100 °C. The poly (TA) rhombus‐like solid product was collected after centrifugation and washed with water for three times, and obtained product was dried at 60 °C in oven and denoted as PTA. The defective graphitic nanosheets were obtained after carbonization of PTA at different temperatures for 3 h with a heating rate of 1 °C min^−1^ under N_2_, denoted as DGN‐T (*T* = 800 °C, 900 °C, and 1000 °C). For comparison, TA was directly pyrolyzed into carbon at 900 °C, resulting in TA‐C.

### Synthesis of KPB

KPB was prepared by a facile precipitation method in an aqueous solution. Firstly, K_4_Fe(CN)_6_ (1 mmol) was dissolved in deionized water (160 mL) to form solution A, and FeCl_3_ (2 mmol) was dissolved in deionized water (40 mL) to form solution B at room temperature. Second, solution B was dropwise added into solution A under stirring and precipitation occurred immediately. The mixture was stirred for 2 h and aged for another 24 h. The obtained dark blue precipitate was collected by centrifugation, washed by water and ethanol, and dried at 80 °C in a vacuum oven all night.

### Materials Characterizations

The morphologies were investigated by SEM (Hitachi SU8010), AFM (JEOL JSPM‐5200), and field emission transmission electron microscope (FE‐TEM, G2 60–300, FEI) equipped with EDS (Super‐X). The chemistry information was examined by XRD (Bruker D2 Phaser X‐Ray Diffractometer with Cu K*α* radiation) and Raman spectroscopy (a Kr‐Ar ion laser at 633 nm produced by Spectra‐Physics Beamlok 2060‐RS laser combining Symphony CCD‐1LS detection system). The nitrogen adsorption–desorption isotherms were measured with a Quantachrome Autosorb AS‐6B system. The near surface chemical state of different elements was also done via XPS (ESCALAB 250 Xi). TGA was conducted by SDT Q600 (TA Instruments) in air with a ramp rate of 10 °C min^−1^.

### Electrochemical Measurements

The anode was prepared by mixing the active materials, carbon black, and polyvinylidene difluoride (PVDF) with a weight ratio of 7:2:1 in *N*‐methyl‐2‐pyrrolidone (NMP). Subsequently, the formed slurry was directly coated onto aluminum foils and dried at 80 °C under vacuum overnight. 2032‐type coin cells (CR2032) were assembled in an Ar‐filled glove box, using the prepared anodes with a mass loading of 1.1–1.3 mg cm^−2^ as work electrode, and potassium metal foils counter electrode. Glass microfiber filters (Whatman, Grade GF/D) and 1.0 m KFSI in DME were used as separators and electrolyte, respectively. And the dosage of electrolyte for each coin cell was around 125 µL. Galvanostatic charge and discharge tests were conducted by multichannel land battery test system (LAND‐CT2001A) in the fixed voltage window from 0.01 to 2.5 V versus K^+^/K. CV and EIS measurements were obtained on a Gamry electrochemical workstation. As for ex situ material characterizations, coin cells were cycled and dissembled in a glove box. These electrodes were used for ex situ measurements after washing with DEC solvent to remove impurities.

Besides, the potassium‐ion full cells were assembled using DGN‐900 as anode and KPB as cathode, with the same separator and electrolyte. In particular, the cathode electrode was prepared in the same way as anode on an aluminum foil. The mass loading of the KPB cathode was approximately four times larger than that of the DGN‐900 anode. Before the assembly of full cell, the DGN‐900 anode and KPB cathode were prepotassiated by charging–discharging for five cycles, respectively.

## Conflict of Interest

The authors declare no conflict of interest.

## Supporting information

Supporting Information

## Data Availability

Research data are not shared.
