# Supplementary material for: Rationally Tailoring Superstructured Hexahedron Composed of Defective Graphitic Nanosheets and Macropores: Realizing Durable and Fast Potassium Storage
Source: Adv Sci (Weinh). 2022 Nov 24;10(3):2205234. doi: 10.1002/advs.202205234 (PMC9875633; doi:10.1002/advs.202205234)
Supplement: Supplementary file 1 — Supporting Information [file ADVS-10-2205234-s001.pdf]

Supporting Information for

Rationally tailoring superstructured hexahedron composed of defective graphitic nanosheets and macropores: Realizing durable and fast potassium storage

Fei Yuan [a], Conghao Shi [a], Yanan Li [a], Jian Wang [a], Di Zhang [a], Wei Wang [b], Qiujun Wang [a], Huan Wang [a], Zhaojin Li [a,] *, Bo Wang [a,] *

[a] *Hebei Key Laboratory of Flexible Functional Materials, School of Materials Science and Engineering, Hebei University of Science and Technology, Shijiazhuang 050000, China*

[b] *School of Metallurgical and Ecological Engineering, University of Science and Technology Beijing, Beijing 100083, China*

*Corresponding authors.

E-mail addresses: zjli11s@alum.imr.ac.cn (Z. L), wangbo@hebust.edu.cn (B. W).

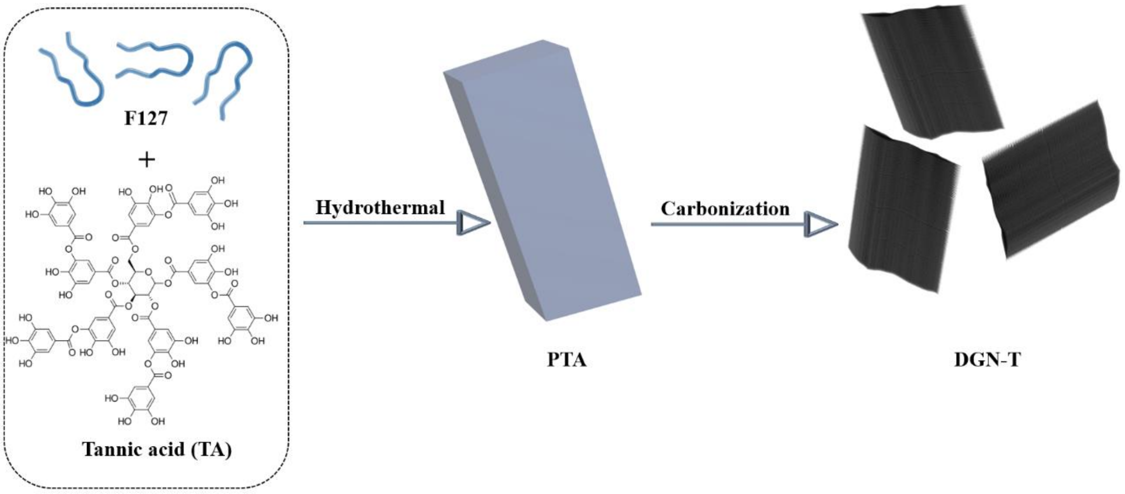

Figure S1. Synthesis process of DGN-T.

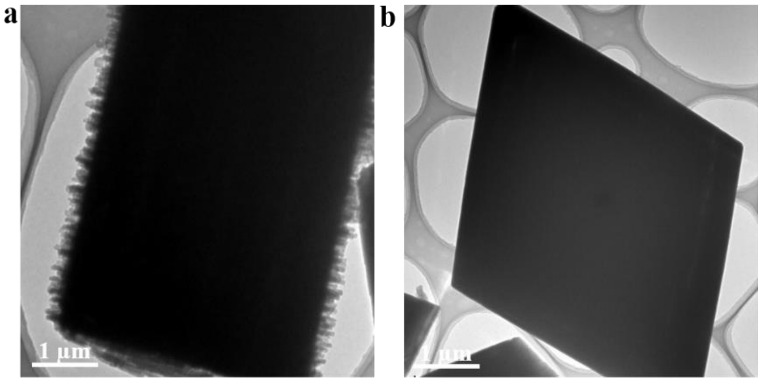

Figure S2. TEM images of PTA.

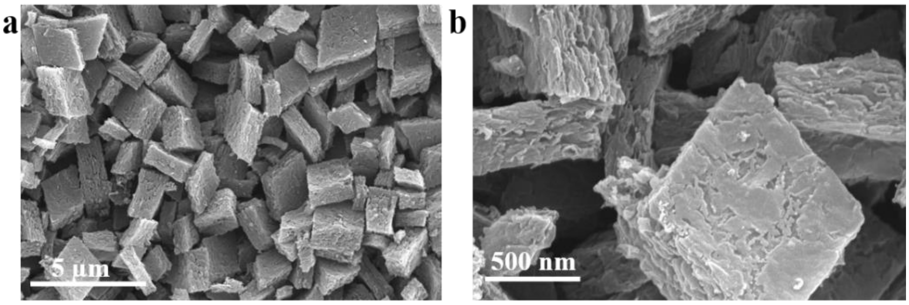

Figure S3. SEM images of DGN-900.

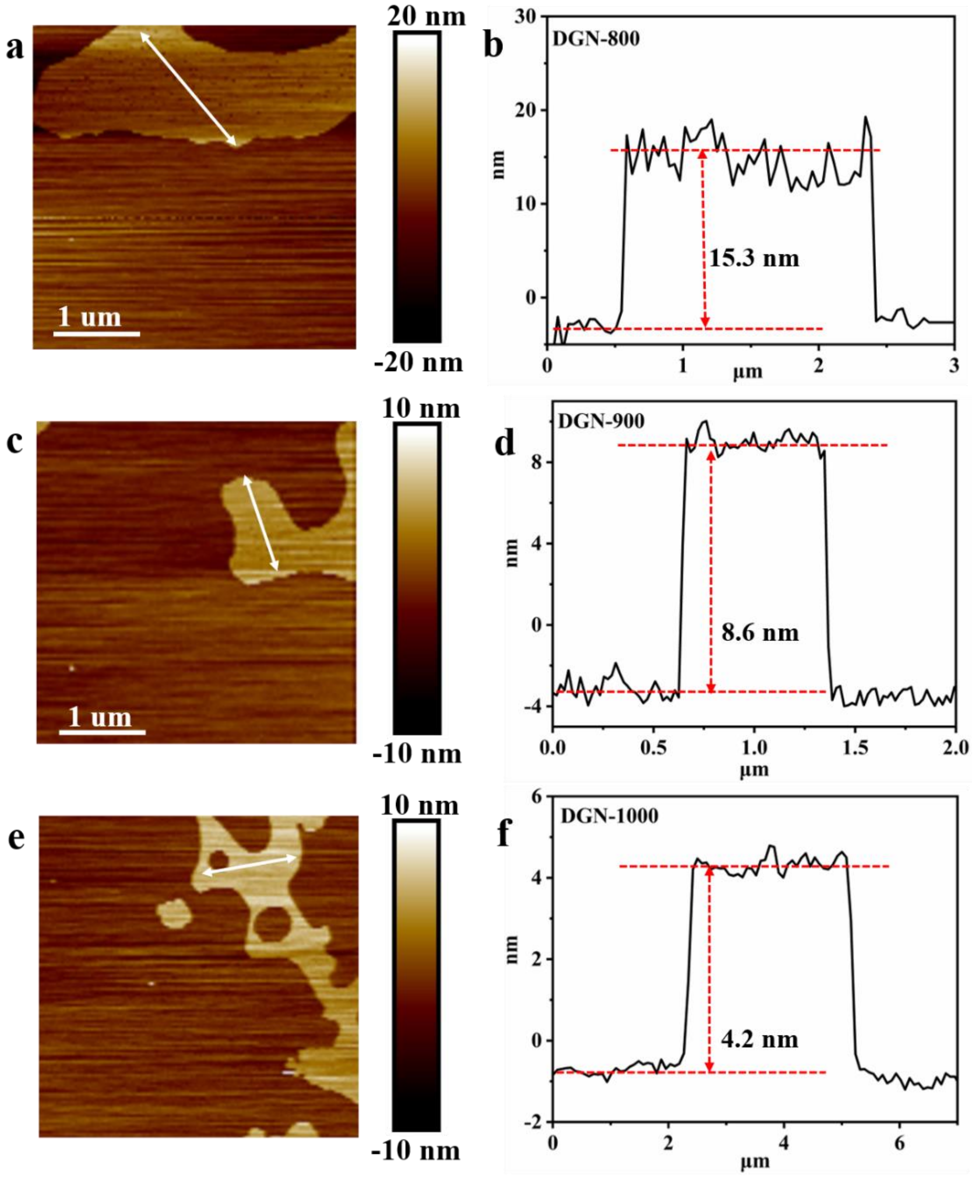

Figure S4. AFM images and the corresponding line scans analysis of (a, b) DGN-800, (c, d) DGN-900, and (e, f) DGN-1000, respectively.

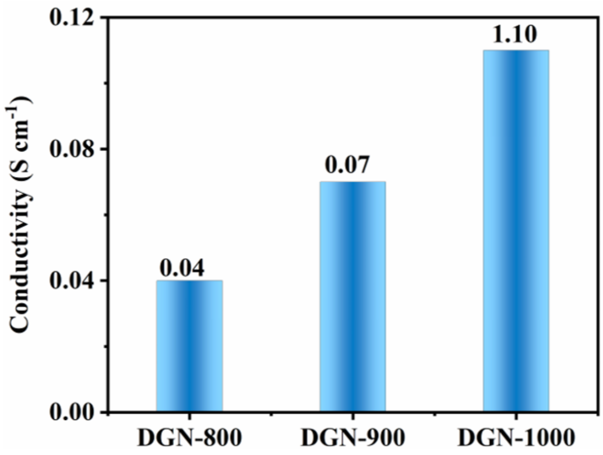

Figure S5. Electronic conductivity measurements of DGN-800, DGN-900, and DGN-1000.

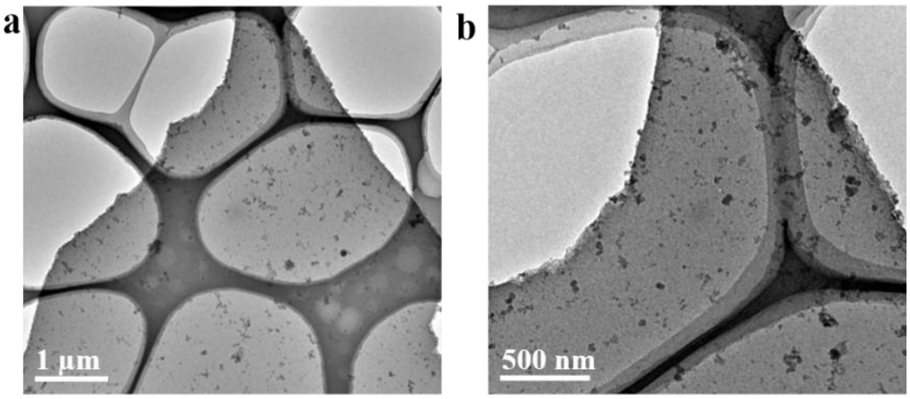

Figure S6. TEM images of TA-C.

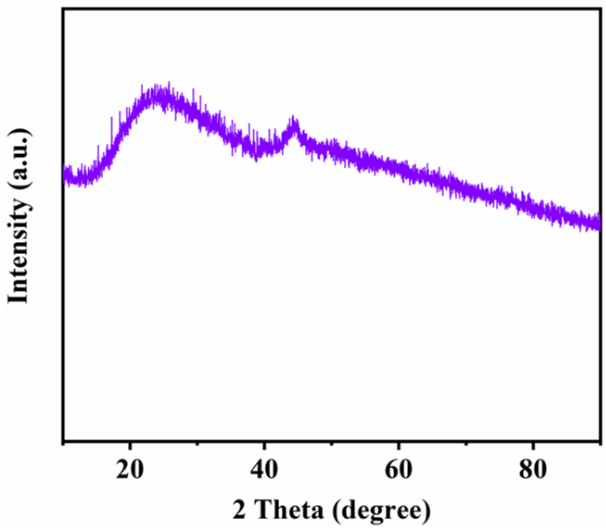

Figure S7. XRD pattern of TA-C.

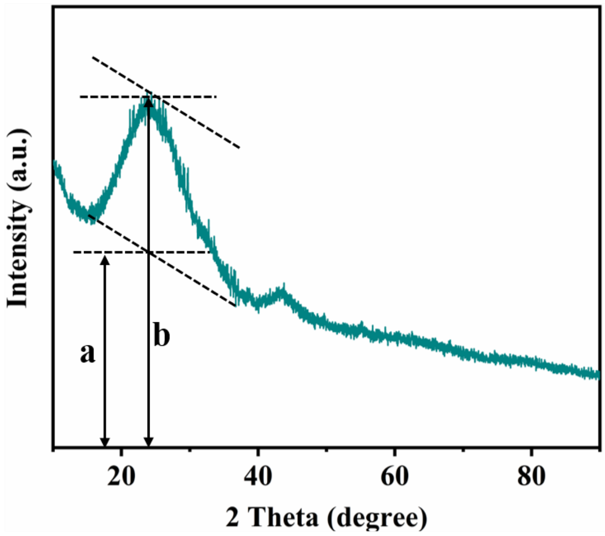

Figure S8. XRD patterns for calculating R value.

The R value is determined by the ratio of *b* to *a* where *b* corresponds to the height of the longer line with arrow, and *a* corresponds to the height of the shorter line with arrow. To obtain the value of *a* and *b*, *a* straight line to connect the two sides of the (002) peak was firstly drawn. Then, another straight line was established by making it parallel with above line while tangent to the (002) peak. Meanwhile, drawing a line with arrow via the point of tangency was vertical to the *x* axis. This line was separated

into two parts by the first drawn line. And the length of this line was the value of *b*, the length of the part close to x axis was the value of *a*.

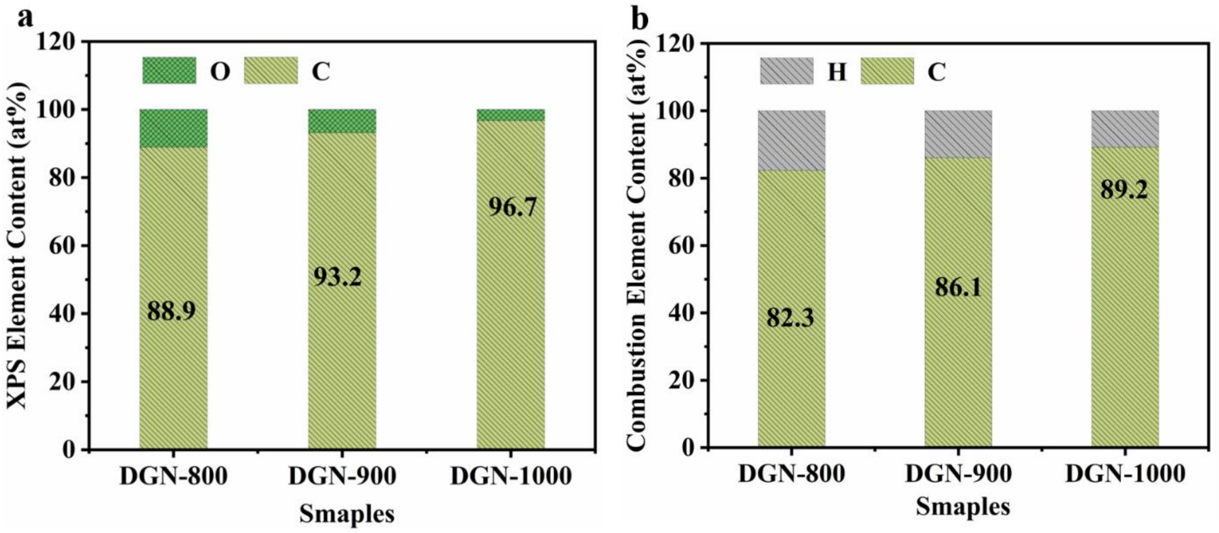

Figure S9. Element content of C and O for DGN-800, DGN-900, and DGN-1000 based on the (a) XPS and (b) combustion analysis.

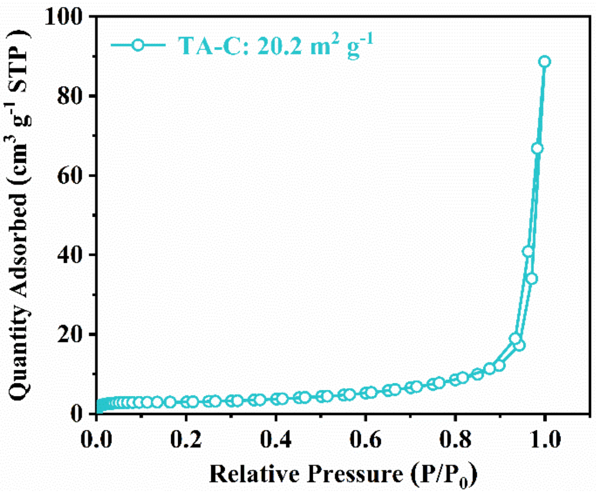

Figure S10. Nitrogen-adsorption/desorption isotherms of TA-C.

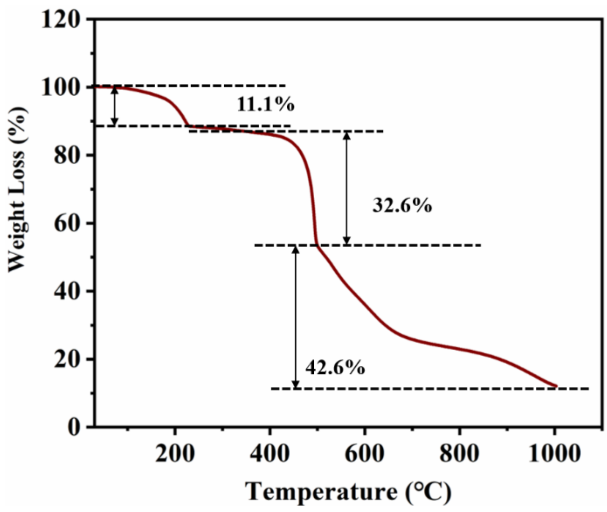

Figure S11. TG curves of PTA.

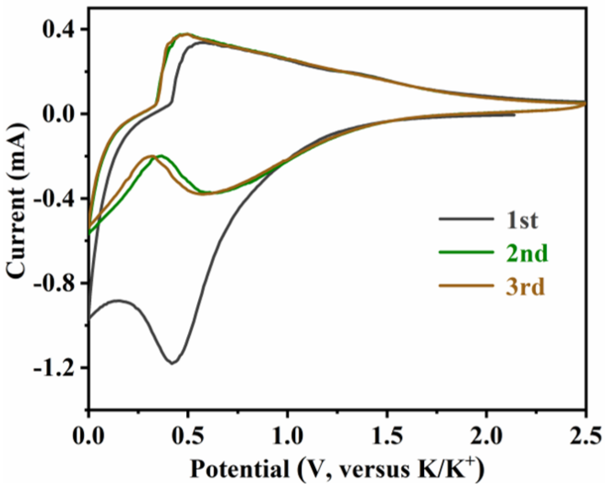

Figure S12. CV curves of DGN-900.

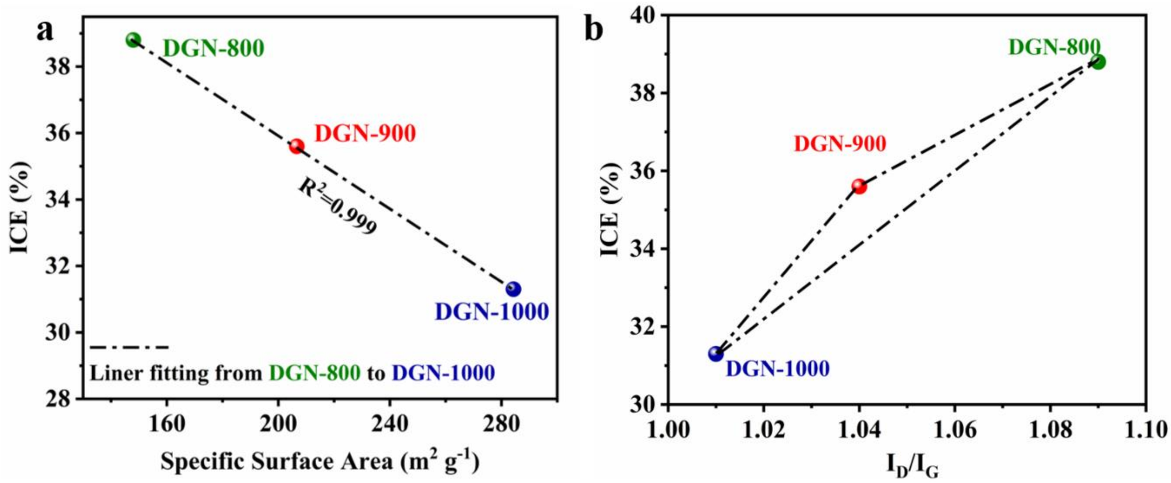

Figure S13. (a) Correlation between ICE and specific surface area. (b) Correlation between ICE and I_D/I_G.

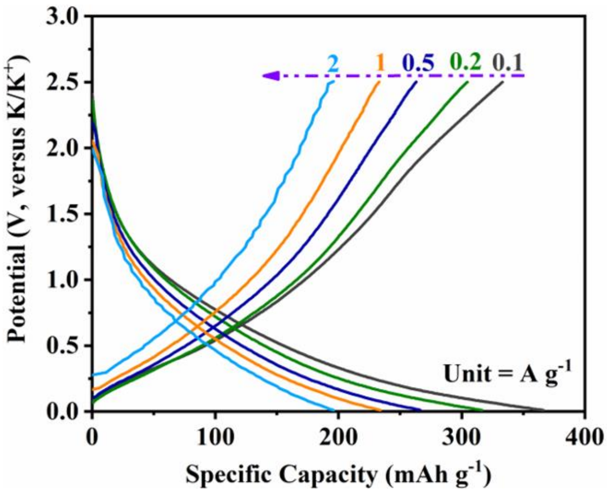

Figure S14. Charge/discharge curves of DGN-900 at different current densities.

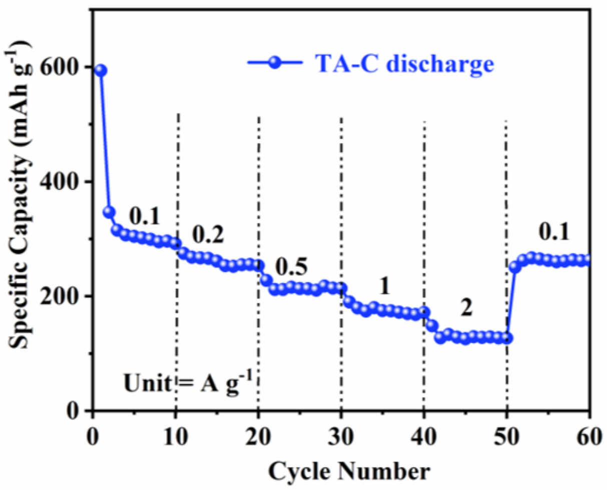

Figure S15. Rate capabilities of TA-C at different current densities.

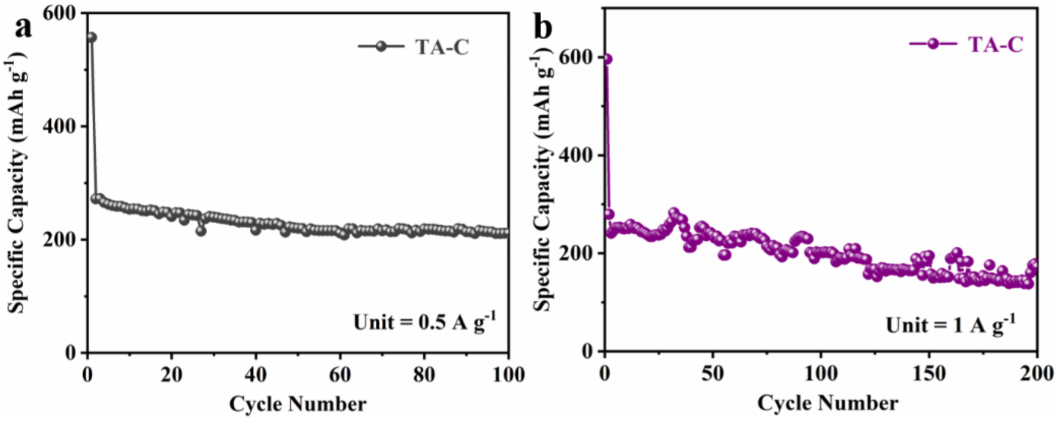

Figure S16. Cycling performance of TA-C at (a) 0.5 and (b) 1 A g^{-1}, respectively.

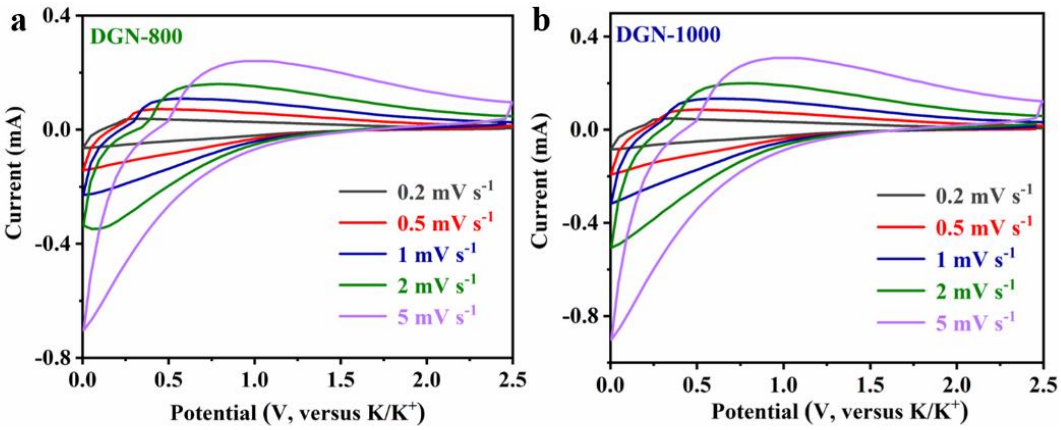

Figure S17. CV curves of (a) DGN-800 and (b) DGN-1000 at different scan rates, respectively.

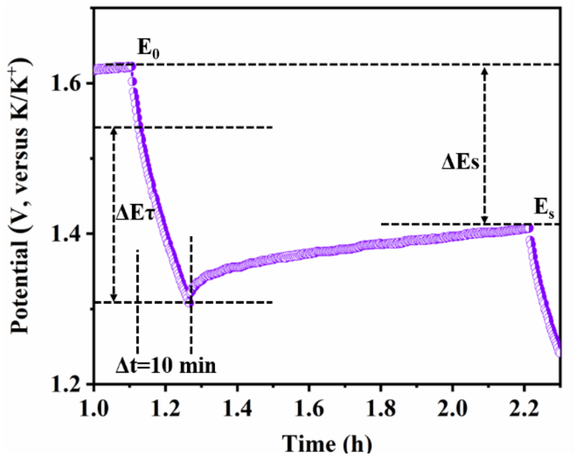

Figure S18. Schematic of the calculation of diffusion coefficient using GITT technique.

The ionic diffusion coefficient in DGN-800, DGN-900, and DGN-1000 electrodes can be determined by solving Fick's second law with the following equation

$$D=\frac{4}{\pi\tau}\left(\frac{m_B V_M}{M_B S}\right)^2\left(\frac{\Delta E_S}{\Delta E_\tau}\right)^2$$

where m_B is electrode active mass; M_B is the molar mass of the electrode material for carbon; V_M is the molar volume of hard carbon; S is the geometric area of the electrode; M_B/V_M is obtained from the density of DGN-800, DGN-900, and DGN-1000.

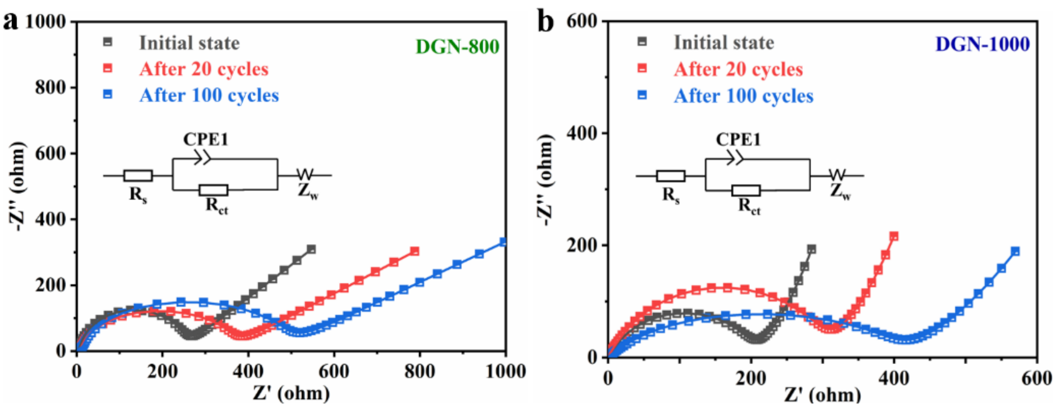

Figure S19. Nyquist plots of (a) DGN-800 and (b) DGN-1000 before and after cycling.

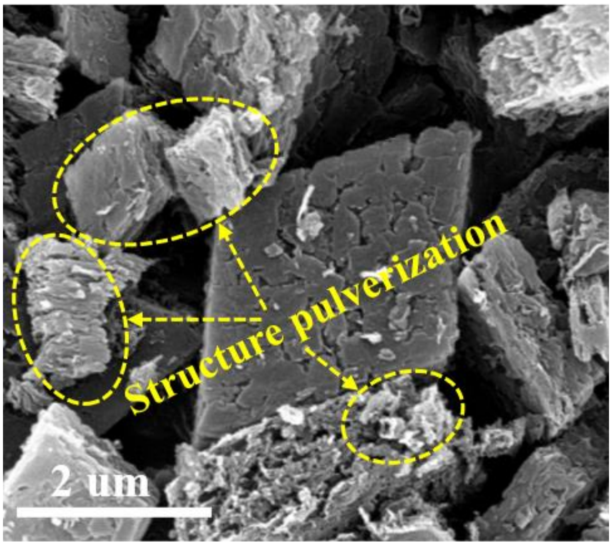

Figure S20. SEM image of DGN-900 after 2000 cycles at 2 A g^{-1}.

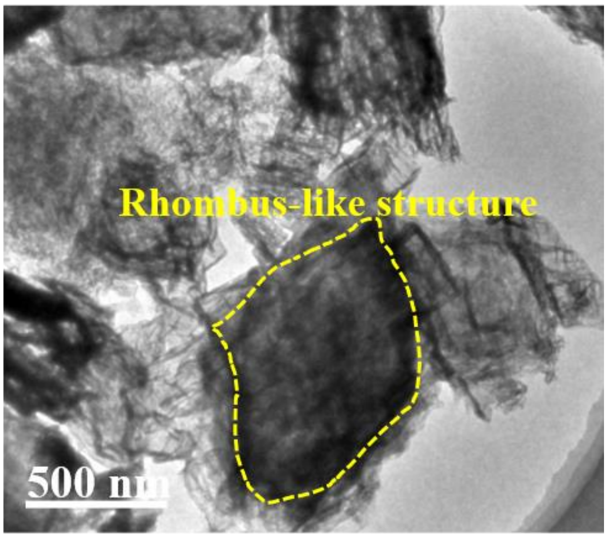

Figure S21. TEM image of DGN-900 after 2000 cycles at 2 A g^{-1}.

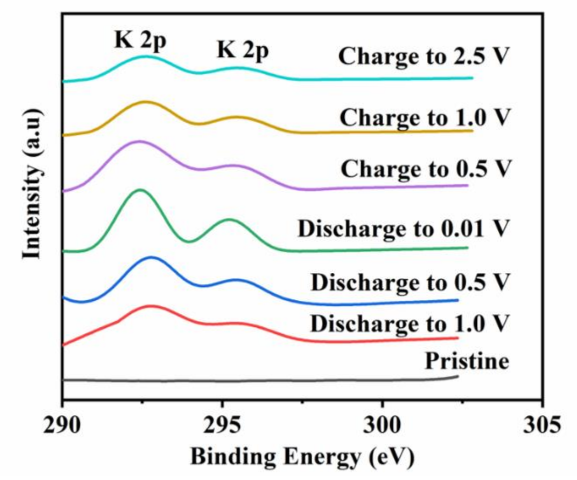

Figure S22. K 2p high-resolution XPS spectra of DGN-900 during the initial discharging/charging.

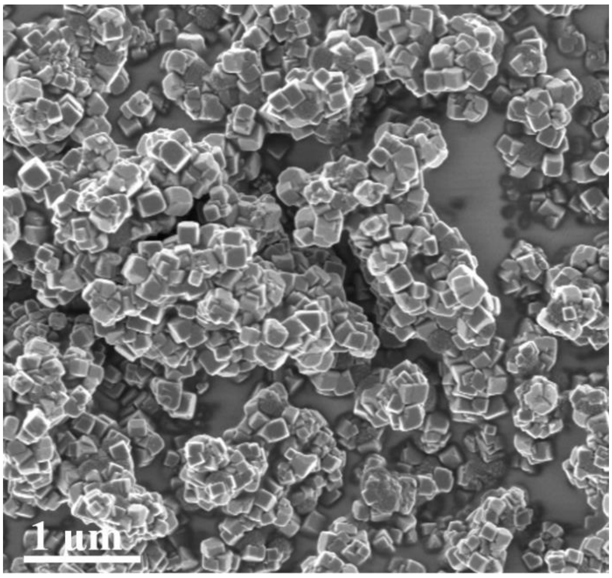

Figure S23. SEM image of KPB.

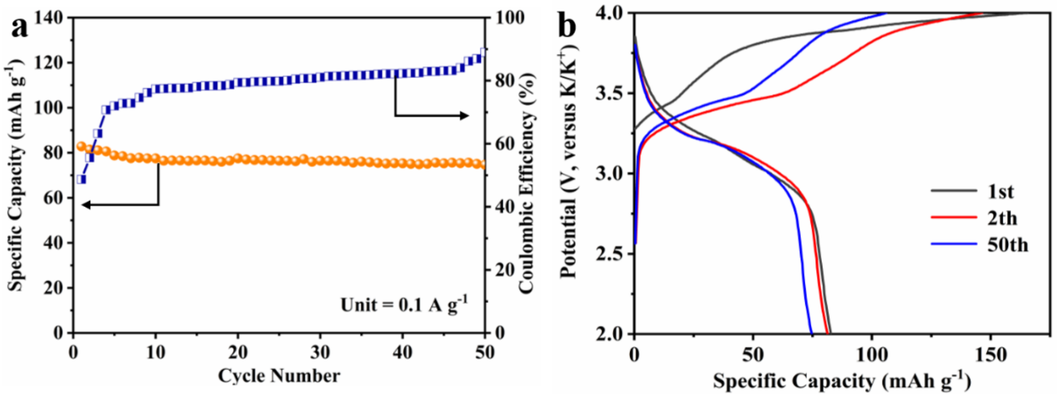

Figure S24. (a) Cycling performance and (b) galvanostatic charge/discharge profiles of KPB at 0.1 A g^{-1} in a voltage window of 2-4 V.

Table S1. Pore structure parameters of the prepared carbon material.

**Materials**	**S_{BET} (m^2 g^{-1})**	**Micropores Volume (cm^3 g^{-1})**	**Mesopores Volume (cm^3 g^{-1})**	**Macropores Volume (cm^3 g^{-1})**
DGN-800	148	0.0720	0.4954	-
DGN-900	206.6	0.5329	1.431	25.2
DGN-1000	284.3	1.134	1.7878	2.88

Table S2. Comparison of carbon-based anodes in potassium-ion batteries

Material name	Cycled capacity (mAh g^{-1})/current density (A g^{-1})	Cycle number	Ref.
**DGN-900**	**193.5/2**<br>**218.9/1**	**2000**<br>**500**	**This work**
N/F-doped soft carbon	186/1	1000	Ref. [1]
meso-C 1st	133/1	1000	Ref. [2]
MCS-7-900	169.6/2	1000	Ref. [3]
Nitrogen-doped carbon	143/1	2000	Ref. [4]
Hard carbon porous nanobelts	277/1	1600	Ref. [5]
3D nitrogen-doped turbostratic carbon	200/1	500	Ref. [6]
Nitrogen/sulfur co-doping carbon	105.2/	600	Ref. [7]
S/N co-doping Graphene nanosheets	188.8/1	2000	Ref. [8]
Nitrogen-doped soft carbon	165/1	500	Ref. [9]
Nitrogen/phosphorus dual-doped porous carbons	153/1	400	Ref. [10]
N/S/O tri-doped hard carbon	136/0.5	200	Ref. [11]

References

[1] Y. Zhong, W. Dai, D. Liu, W. Wang, L. Wang, J. Xie, R. Li, Q. Yuan, G. Hong, Nitrogen and fluorine dual doping of soft carbon nanofibers as advanced anode for potassium ion batteries, Small 17 (2021) 2101576.

[2] R. Guo, X. Liu, B. Wen, F. Liu, J. Meng, P. Wu, J. Wu, Q. Li, L. Mai, Engineering mesoporous structure in amorphous carbon boosts potassium storage with high initial Coulombic efficiency, Nano-Micro Lett. 12 (2020) 148.

[3] J. Zheng, Y. Wu, Y. Tong, X. Liu, Y. Sun, H. Li 1, L. Niu, High Capacity and fast kinetics of potassium-ion batteries boosted by nitrogen-doped mesoporous carbon spheres, Nano-Micro Lett. 13 (2021) 174.

[4] J. Li, Y. Li, X. Ma, K. Zhang, J. Hu, C. Yang, M. Liu, A honeycomb-like nitrogen-doped carbon as high-performance anode for potassium-ion batteries, Chem. Eng. J. 384 (2020) 123328.

[5] K. Zhang, Q. He, F. Xiong, J. Zhou, Y. Zhao, L. Mai, L. Zhang, Active sites enriched hard carbon porous nanobelts for stable and high-capacity potassium-ion storage, Nano Energy 77 (2020) 105018.

[6] W. Zhang, J. Yin, M. Sun, W. Wang, C. Chen, M. Altunkaya, A. Emwas, Y. Han, U. Schwingenschlögl, H. N. Alshareef, Direct pyrolysis of supermolecules: An ultrahigh edge-nitrogen doping strategy of carbonanodes for potassium-ion batteries, Adv. Mater. 32 (2020) 2000732.

[7] X. Shi, Y. Zhang, G. Xu, S. Guo, A. Pan, J. Zhou, S. Liang, Enlarged interlayer spacing and enhanced capacitive behavior of a carbon anode for superior potassium storage, Sci. Bull. 65 (2020) 2014-2021.

[8] W. Yang, J. Zhou, S. Wang, Z. Wang, F. Lv, W. Zhang, W. Zhang, Q. Sun, S. Guo, A three-dimensional carbon framework constructed by N/S co-doped graphene nanosheets with expanded interlayer spacing facilitates potassium ion storage, ACS Energy Lett. 5 (2020) 1653−1661.

[9] C. Liu, N. Xiao, H. Li, Q. Dong, Y. Wang, H. Li, S. Wang, X. Zhang, J. Qiu, Nitrogen-doped soft carbon frameworks built of well-interconnected nanocapsules enabling a superior potassium-ion batteries anode, Chem. Eng. J. 382 (2020) 121759.

[10] X. Ma, N. Xiao, J. Xiao, X. Song, H. Guo, Y. Wang, S. Zhao, Y. Zhong, J. Qiu, Nitrogen and phosphorus dual-doped porous carbons for high-rate potassium ion batteries, Carbon 179 (2021) 33-41.

[11] M. Chen, Y. Cao, C. Ma, H. Yang, A N/S/O-tridoped hard carbon network anode from mercaptan/polyurethane-acrylate resin for potassium-ion batteries, Nano Energy 81 (2021) 105640.

Table of Contents

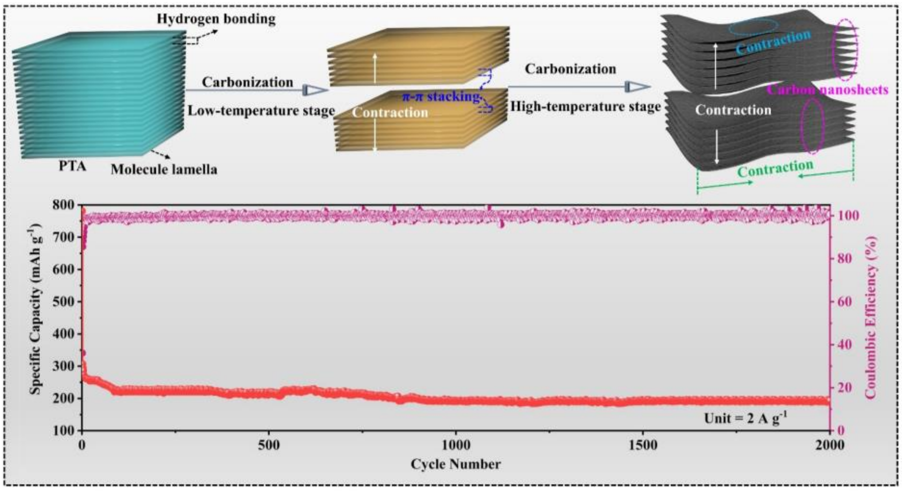

Due to the spatially confined two-step local contraction of poly tannic acid (PTA) along different directions and dimensions during carbonization, defective nanosheets with macropores are formed, while realizing a balance between defects content and graphitization degree by regulating temperature. All of which endow the fast transfer ability of both ions/electrons, and alleviate volume fluctuation, leading to improved capacity and cyclability.
